# The challenges of estimating the prevalence of child maltreatment

**DOI:** 10.1186/1745-6215-16-S2-P166

**Published:** 2015-11-16

**Authors:** Gwenllian Moody, Michael Robling, Kerenza Hood, Rebecca Cannings-John, Alison Kemp

**Affiliations:** 1 Cardiff University, Cardiff, UK

## Aims

Counting a condition that has no gold standard diagnostic test such as child maltreatment presents challenges. This literature review aims to review both formally collected statistics from agencies in the UK, and studies using various methodologies to capture prevalence rates in the UK and worldwide.

## Methods

PubMed, Ovid SP and grey literature from the NSPCC, UNICEF, The UK Government, and WHO were reviewed from 1989 to 2014. A ‘snowballing’ technique was used to identify additional studies. The literature review focused on the various ways maltreatment data are collected in the UK from official statistics, to the variation found in self-reported prevalence of maltreatment between studies worldwide, and how methodological differences may explain differences found in the quoted prevalence figures.

## Results

Official statistics of maltreatment in the UK are only a portion of the true cases. Self-reported prevalence rates are also used to collect data, however, rates vary between studies. Studies conducted in the UK report that between 0.8% to 25% of children have a lifetime prevalence rate of maltreatment (sexual, physical, and emotional abuse, and neglect), studies conducted worldwide showed even greater variation of between 0.2% and 77%.

## Discussion

Official statistics on maltreatment include only the tip of the iceberg of true cases. Self-report studies can also be used to estimate maltreatment, however, prevalence rates differ vastly between studies, this is likely due to methodological differences, which include the data source, the study participants, and the definition used of maltreatment.

